# Mononeuritis Multiplex As the Initial Presentation of Eosinophilic Granulomatosis With Polyangiitis (EGPA) in a Non-asthmatic Filipino Female: A Case Report

**DOI:** 10.7759/cureus.82159

**Published:** 2025-04-13

**Authors:** Fallen Grace E De la Paz, Ludwig F Damian

**Affiliations:** 1 Neurology, St. Luke's Medical Center, Quezon City, PHL

**Keywords:** egpa, filipino, mononeuritis multiplex, nonasthmatic, treatment outcome

## Abstract

Eosinophilic granulomatosis with polyangiitis (EGPA) is a rare disease characterized by necrotizing vasculitis of small and medium-sized systemic blood vessels. The three phases to this disease are: 1) prodromal phase, which includes asthma, 2) eosinophilic phase, and 3) vasculitic phase. There is currently no diagnostic criterion for the diagnosis of EGPA, and diagnosis relies more on clinical features. On laboratory testing, peripheral eosinophilia is a hallmark of diagnosis. Treatment for new-onset antineutrophil cytoplasmic antibody (ANCA)-associated vasculitis with organ or life-threatening disease includes a combination of rituximab with glucocorticoids. This paper presents the case of a 40-year-old non-asthmatic female from the Philippines who initially presented with subacute progressive asymmetric bilateral leg pain with distal weakness and numbness. Blood tests for complete blood count, antineutrophil cytoplasmic antibodies, and inflammatory markers were done along with nerve conduction studies. Diagnostics revealed eosinophilia, perinuclear anti-neutrophil cytoplasmic antibodies positivity, and mononeuritis multiplex, which led to the diagnosis of EGPA. She was treated with steroids and rituximab. A follow-up after one year of treatment showed marked improvement of neurologic status and functional outcome. Muscle strength and sensation had improved, and the patient was eventually able to ambulate with minimal assistance and work on a computer job. This case reports atypical presentation of EGPA, initially presenting as malaise and sensorimotor disturbances with gradual progression of symptoms in four weeks with no history of severe asthma or any respiratory symptoms.

## Introduction

The most common primary systemic small-vessel vasculitis in adults is the antineutrophil cytoplasmic antibody (ANCA) associated vasculitis. The clinical manifestations of these types of vasculitis involve different organ systems. Skin lesions will present as palpable purpura, and neurologic symptoms will present as peripheral neuropathy such as mononeuritis multiplex. Constitutional symptoms include fever, weight loss, anorexia, and general malaise. A type of small vessel ANCA-associated vasculitis is eosinophilic granulomatosis with polyangiitis (EGPA) [1]. EGPA affects up to three per million adults worldwide. It is characterized by necrotizing vasculitis of small and medium-sized systemic blood vessels. The clinical course of EGPA is characterized by three phases. The prodromal phase is characterized by atopy, allergic rhinitis, and asthma. Asthma is often adult-onset and severe, present in at least 96% of patients. This phase also includes malaise, fever, migrating polyarthralgia, and weight loss. The second phase is the eosinophilic infiltration in end organs and peripheral eosinophilia. The third phase is the onset of vasculitis, which is usually hallmarked by neurologic symptoms, most commonly mononeuritis multiplex or mixed sensorimotor polyneuropathy. The central nervous system can also be affected in up to 39% [2]. Some studies state that the vasculitic phase usually develops within three years of the onset of asthma [1]. However, these phases may overlap with one another [2].

The pathogenesis and clinical phenotype of EGPA are due to either eosinophil-mediated damage or ANCA-induced endothelial injury. Eosinophils release proteins that are directly involved in mediating tissue damage. Peripheral eosinophilia is a hallmark of diagnosis, and the degree of eosinophilia is associated with the severity of vasculitis [2]. Significant pathologies for EGPA include eosinophilic inflammatory infiltrates, extravascular granulomas, and necrotizing vasculitis or pauci-immune necrotizing glomerulonephritis. Biopsy may be done on purpuric lesions, kidneys, or the lungs and is most definitive for the confirmation of disease. However, it is not required for diagnosis [3]. On other workup, the most frequent type of antibody found in EGPA is perinuclear ANCAs (p-ANCAs) on indirect immunofluorescence test [4]. P-ANCA sensitivity is 46.3% and specificity is 91.4% for ANCA-associated vasculitis [5]. The p-ANCA, aimed toward myeloperoxidase (MPO-ANCA), often appears in patients suffering from microscopic polyangitis or EGPA. In patients with a compatible clinical phenotype, ANCA positivity supports the diagnosis of EGPA. To date, there are no systematically developed evidence-based guidelines dedicated to the diagnosis of EGPA [6]. In research, there is a classification criterion by the 2022 American College of Rheumatology/European Alliance of Associations for Rheumatology for EGPA. Included in the criteria is the presence of a blood eosinophil count ≥1×10^9^/liter, scoring 5 points, and mononeuritis multiplex/motor neuropathy, scoring 1 point. A score of 6 and above is required. The sensitivity is 85% and the specificity is 99% [7]. The diagnosis, however, still relies more on clinical features rather than histopathology or laboratory testing [2]. Treatment for new onset ANCA-associated vasculitis with organ or life-threatening disease includes a combination of cyclophosphamide or rituximab with glucocorticoids [8]. This study presents a case of a 40-year-old non-asthmatic female diagnosed with EGPA with an atypical presentation of mononeuritis multiplex, which is rare. Treatment with prednisone and rituximab was given, and functional status after one year of follow-up was reported. The common initial presentation of the disease will include asthma, atopy, and allergic rhinitis; thus, an atypical presentation may lead to delayed diagnosis or risks of misdiagnosis. The significance of this case includes increased awareness, early diagnosis, and treatment direction for this rare disease. It can also aid in further research of EGPA in Filipinos. No other local reports of similar cases were reported.

## Case presentation

We report the case of a non-asthmatic 40-year-old female, independent in all activities of daily living, who presented with a four-week history of generalized weakness followed by progressive asymmetric bilateral leg pain with distal weakness and numbness prior to hospital consult. This was eventually accompanied by bilateral hand paresthesia and weakness. The symptoms involved different focal nerve territories, such as the right median nerve, left ulnar nerve, and bilateral tibial nerves, on history. Symptoms resulted in impairment of daily living, such as difficulty in texting, writing, and walking. Initially, she was assisted in ambulation. Outpatient consult was done, and she had a complete blood count which showed a white blood cell count of 9,360mm^3^ and eosinophils of 9.3%. She was given pregabalin 150mg once daily and etoricoxib 60mg twice a day as needed for pain, which provided mild relief. The patient later became bed-bound, which prompted the hospital consult. On physical examination, she had purpuric lesions on bilateral arms and legs with bipedal edema. She had a normal temperature, respiratory rate, and cardiac rate with clear breath sounds. Neurological exam showed normal mental status, cranial nerve, and cerebellar tests. Motor testing showed muscle weakness involving the distal extremities more than the proximal extremities. All proximal muscles of bilateral upper extremities, until the elbow extension scored 4+/5 on manual muscle testing. Wrist flexion and extension scored 4/5 on both sides. Finger flexion, adduction, and abduction on the first to third digits scored 2/5 on the right and 3/5 on the left. The finger flexion, adduction, and abduction on the fourth to fifth digit scored 3/5 on the right and 2/5 on the left. Hip flexion, adduction, and abduction scored on both sides scored 4+/5. Bilateral knee flexion and extension scored 4+/5 as well. Ankle dorsiflexion scored 4/5 on bilateral sides. Toe flexion scored 3/5 on the right toe and 4/5 on the left. Ankle plantarflexion, foot eversion, and foot inversion scored 0/5 on the right and 1/5 on the left. The lower extremities were more affected than the upper extremities. The right extremities were more affected than the left extremities. Asymmetrical sensory loss on the distal bilateral lower extremities was also noted. The pattern of the sensory loss initially started on the palmar and solar aspects. Physical examination showed that the last normal sensory level was at the bilateral legs. All areas more distal had decreased sensation on pinprick, light touch, temperature, vibration, and proprioception. Bilateral dorsum had 95% intact sensation, bilateral medial malleolus and medial foot had 80% intact sensation, bilateral lateral malleolus to the lateral foot had no sensation, left sole had 20% intact sensation, and the right sole had no sensation. This showed a peripheral nerve involvement. There was the presence of mechanical allodynia and hyporeflexia on the right patella and bilateral Achilles tendons. Patient tested negative for Babinski reflex. Autonomic function remained intact.

Laboratory results during admission revealed eosinophilia, elevated erythrocyte sedimentation rate, antinuclear antibody (ANA) positive at 1:640 speckled pattern, and a positive result for p-ANCA via immunofluorescence test. It was not determined if PR3 or MPO type (Table 1).

**Table 1:** Pertinent laboratory results with reference interval p-ANCA, perinuclear anti-neutrophil cytoplasmic antibody; ANA, antinuclear antibody

Laboratory	Result	Reference Range
White blood cell count	9,360 mm^3^	4,800-10,800 mm^3^
Eosinophils	10%	0%-7%
Erythrocyte sedimentation rate	76 mm/hr	0-20 mm/hr
ANA	Positive above 1:640 speckled pattern	Positive/negative
p-ANCA	Positive	Positive/negative

A nerve conduction study done one month after the onset of symptoms showed findings of absent sural, superficial peroneal, and medial plantar sensory potentials. The right tibial motor potentials are too small to be measured. The proximal motor amplitudes of the right median, left ulnar, and left tibial nerves are reduced. The right plantar motor amplitudes are reduced significantly when compared to the left. The tibial F-waves are absent. The H-reflex is absent for both sides. The study confirmed a distal sensory axonal neuropathy along with a patchy, multifocal, motor axonal polyneuropathy with conduction block, consistent with mononeuritis multiplex (Table 2). The patient was diagnosed as EGPA through the clinical features of her disease and the presence of mononeuritis multiplex, eosinophilia, and p-ANCA positivity.

**Table 2:** Nerve conduction studies SAP, sensory action potential; MCV, motor conduction velocity; CMAP, compound muscle action potential; µV, microvolts; m/sec, meters per second; ms, milliseconds; mV, millivolts

	Value	Reference value	Conduction velocity	Reference value
Left median nerve				
SAP (index-wrist)	15.0 µV	>10 µV	55.0 m/sec	> 50 m/sec
SAP (palm-wrist)	75.0 µV	>10 µV	51.0 m/sec	> 44 m/sec
SAP (wrist-elbow)	8.0 µV	>10 µV	66.0 m/sec	> 44 m/sec
MCV	58.0 m/sec	>50 m/sec		
Distal latency	3.4 ms	< 4.2 ms		
CMAP (wrist)	13.7 mV	> 7 mV		
CMAP (elbow)	12.7 mV	> 7 mV		
F-wave to abductor pollicis brevis	24.3 ms	< 40 ms		
Right median nerve				
SAP (index-wrist)	27.0 µV	>10 µV	54.0 m/sec	> 50 m/sec
SAP (palm-wrist)	109.0 µV	>10 µV	50.0 m/sec	> 44 m/sec
SAP (wrist-elbow)	6.0 µV	>10 µV	70.0 m/sec	> 44 m/sec
MCV	47.0 m/sec	>50 m/sec		
Distal latency	2.8 ms	< 4.2 ms		
CMAP (wrist)	4.4 mV	> 7 mV		
CMAP (elbow)	1.5 mV	> 7 mV		
F-wave to abductor pollicis brevis	25.1 ms	< 40 ms		
Left ulnar nerve				
SAP (digit 5 wrist)	20.0 µV	> 7 µV	57.0 m/sec	> 48m/sec
MCV (forearm)	54.0 m/sec	> 50 m/sec		
MCV (across elbow)	62.0 m/sec	> 50 m/sec		
Distal latency	2.7 ms	< 4.2 ms		
CMAP (wrist)	8.8 mV	> 5 mV		
CMAP (elbow)	5.1 mV	> 5 mV		
CMAP (above elbow)	4.8 mV	> 5 mV		
F-wave to abductor digiti minimi	26.9 ms	< 40 ms		
Right ulnar nerve				
SAP (digit 5 wrist)	27.0 µV	> 7 µV	56 m/sec	> 48m/sec
MCV (forearm)	61.0 m/sec	> 50 m/sec		
MCV (across elbow)	65.0 m/sec	> 50 m/sec		
Distal latency	2.8 ms	< 4.2 ms		
CMAP (wrist)	11.6 mV	> 5 mV		
CMAP (elbow)	11.6 mV	> 5 mV		
CMAP (above elbow)	11.7 mV	> 5 mV		
F-wave to abductor digiti minimi	24.2 ms	< 40 ms		
Left radial nerve				
SAP	61.0 µV	> 7 µV	64.0 m/sec	> 48 m/sec
Right radial nerve				
SAP	56 µV	> 7 µV	65.0 m/sec	> 48 m/sec
Left sural nerve				
SAP	No response	> 5 mV	No response	> 40 m/sec
Right sural nerve				
SAP	No response	> 5 mV	No response	> 40 m/sec
Left common peroneal				
SAP	No response	> 3 µV	No response	> 40 m/sec
MCV (leg)	50.0 m/sec	> 40 m/sec		
MCV (across leg)	60.0 m/sec	> 40 m/sec		
Distal latency	3.3 ms	< 5.5 ms		
CMAP (ankle)	2.5 mV	> 3 mV		
CMAP (knee)	2.4 mV	> 3 mV		
CMAP (above knee)	2.4 mV	> 3 mV		
Right common peroneal				
SAP	No response	> 3 µV	No response	> 40 m/sec
MCV (leg)	49.0 m/sec	> 40 m/sec		
MCV (across leg)	57.0 m/sec	> 40 m/sec		
Distal latency	2.5 ms	< 5.5 ms		
CMAP (ankle)	5.5 mV	> 3 mV		
CMAP (knee)	4.4 mV	> 3 mV		
CMAP (above knee)	4.0 mV	> 3 mV		
Left posterior tibial				
MCV	45.0 m/sec	> 40 m/sec		
Distal latency	2.9 ms	< 6 ms		
CMAP (ankle)	12.4 mV	> 6 mV		
CMAP (knee)	4.8 mV	> 6 mV		
F-wave to abductor hallucis	No clear F-wave	< 60 µV		
H-reflex to gastroc soleus	No response	+1		
Right posterior tibial				
MCV	-	> 40 m/sec		
Distal latency	4.8 ms	< 6 ms		
CMAP (ankle)	0.6 mV	> 6 mV		
CMAP (knee)	No response	> 6 mV		
F-wave to abductor hallucis	Extremely low amplitude	< 60 µV		
H-reflex to gastroc soleus	No response	+1		
Left medial plantar nerve				
SAP	No response	> 5V		
Right medial plantar nerve				
SAP	No response	> 5 µV		
Left lateral plantar nerve				
SAP	No response	> 5 µV		
Right lateral plantar nerve				
SAP	No response	> 5 µV		
Left plantar nerve				
Latency	6.2 ms	=/< 3.8 ms		
Amplitude	5.7 mV	> 3 mV		
Right plantar nerve				
Latency	4.4 ms	=/< 3.8 ms		
Amplitude	0.2 mV	> 3 mV		

She was treated with prednisone initially given at 50mg/day and was tapered down 5mg every five days and eventually discontinued. Patient was not given intravenous (IV) steroids due to financial difficulties with the additional costs of prolonging hospital stay. She opted to continue oral medications at home. She eventually underwent three cycles of rituximab as outpatient in a span of one year. The first cycle of rituximab was given 1g/IV at two weeks apart, then 500mg/IV every six months for two more cycles. Ideally, the second and third cycles were to be given at 1g/IV; however, due to financial issues, she was only given 500mg/IV. She was given a total of 3g of rituximab. During the course of the treatment, she had regular follow-up with her rheumatologist and neurologist. The patient had multiple tests done for complete blood count, urinalysis, creatinine and liver enzymes in order to check for any infection and derangements in renal and liver function which were unremarkable. Her repeat erythrocyte sedimentation rate done interim had decreased to 16mm/hr and eosinophils were at 2.6% of a white blood cell count of 7,300mm^3^. The patient had marked improvement after a year of treatment. Ambulation was still assisted due to residual weakness of bilateral lower extremities, but she was now able to work on computer jobs with upper extremity weakness fully resolved.

## Discussion

The atypical initial presentation of the patient was malaise and sensorimotor disturbances with gradual progression of symptoms in four weeks, with no history of severe asthma or any respiratory symptoms. In EGPA, the neurologic symptoms are part of the vasculitic stage and are not usually apparent at the beginning of the disease. This makes diagnosis particularly challenging, especially in the absence of respiratory symptoms that are more typical of EGPA [9]. ANAs and ANCA were requested due to the high index of suspicion of an autoimmune disease despite the absence of asthma. The workup revealed that she had positive ANA, p-ANCA positivity, eosinophilia, and mononeuritis multiplex. The diagnosis was made due to the clinical features of the patient with the support of the laboratory findings. To date, there are no diagnostic criteria for the diagnosis of EGPA [6]. The patient did not fulfil the American College of Rheumatology/European Alliance of Associations for Rheumatology criteria for EGPA since her eosinophil count was only 0.9×10^9^/liter. However, she still presented with eosinophilia, a hallmark of this disease, which distinguishes it from other ANCA-associated vasculitis [2].

Other studies report on non-asthmatic patients who were diagnosed with EGPA. A study by Kobayashi et al. discussed a woman with a fever of unknown origin with purpura and jaw claudication. She had no history of asthma but with noted eosinophilia and was diagnosed as EGPA with tissue biopsy [10]. Another case report described an elderly woman with worsening renal function preceded by fever, purpura, and sinusitis with eosinophilia on workup. Kidney biopsy showed crescent formations with diffuse interstitial fibrosis and eosinophil infiltration. She was diagnosed with EGPA due to tissue eosinophilia, peripheral eosinophilia, and sinusitis [11]. Similar to the cases presented, the patient did not present with asthma; however, other clinical manifestations were suggestive of EGPA. Although asthma is present in up to 96% of EGPA patients [2], these reports support that not all EGPA patients will present with asthma. There are currently no reports on the prevalence of non-asthmatic EGPA patients.

There are local cases with EGPA presenting as neurologic symptoms. A case report done in the Philippines showed a 40-year-old Filipino male who presented with foot drop diagnosed as Churg-Strauss syndrome, now known as EGPA. He, however, had the typical symptoms of adult-onset asthma and recurrent sinusitis accompanied by dermatologic lesions and arthralgia [12]. Another case report in the Philippines showed a 55-year-old female initially presenting with acute polyneuropathy, eventually diagnosed as EGPA through the American College of Rheumatology 1990 criteria [13]. No other local reports of similar cases were noted. In comparison to other local studies that initially presented with neurologic symptoms at the onset of EGPA, the patient in this case is non-asthmatic.

The patient in our study had early detection of EGPA and had marked improvement after one year of treatment. Prognosis for EGPA is favorable with timely detection and treatment. The five-year survival rate is 90%, and the relapse rate is up to 30%. Prognosis and overall survival improve by early recognition, use of corticosteroids, and immunosuppressants. Corticosteroids inhibit the prolongation of eosinophil survival and reduce eosinophil burden. However, it is also important that the patient is aware of prolonged steroid dependence, and a routine care plan should include screening of glycemic status, bone density, fall prevention, and infection prevention due to possible adverse effects of the medication. [2]. The use of rituximab is effective in remission induction and maintenance treatment of the disease. B cells play a role in the pathogenesis of EGPA, and rituximab is a monoclonal anti-CD20 antibody that depletes B lymphocytes [14].

The documentation of atypical presentations of rare diseases contributes to further the knowledge of the scientific community, especially in the third world setting. This can aid further research and guide therapeutics that may be applied to the same demographics.

## Conclusions

The patient is a 40-year-old female who initially presented with neurologic symptoms and was eventually diagnosed with EGPA due to mononeuritis multiplex, eosinophilia, and p-ANCA positivity. EGPA is challenging to diagnose, especially when it does not present with the typical associated characteristics such as asthma and other respiratory symptoms. Prognosis and overall survival can improve by early recognition and treatment. Cases and treatment outcomes are not well documented in the Philippines, and no other local reports of a similar case were noted. This study can help aid in further research and treatment direction of EGPA in Filipinos.

## References

[1] Mansi IA, Opran A, Rosner F (2002). ANCA-associated small-vessel vasculitis. Am Fam Physician.

[2] Chakraborty RK, Aeddula NR (2025). Eosinophilic Granulomatosis With Polyangiitis (Churg-Strauss Syndrome). https://www.ncbi.nlm.nih.gov/books/NBK537099/.

[3] Wu EY, Hernandez ML, Jennette JC, Falk RJ (2018). Eosinophilic granulomatosis with polyangiitis: clinical pathology conference and review. J Allergy Clin Immunol Pract.

[4] Włudarczyk A, Biedroń G, Wójcik K (2020). ANCA-associated vasculitis patients treated in Polish intensive care units - retrospective characteristics based on the POLVAS registry. Anaesthesiol Intensive Ther.

[5] Guchelaar NA, Waling MM, Adhin AA, van Daele PL, Schreurs MW, Rombach SM (2021). The value of anti-neutrophil cytoplasmic antibodies (ANCA) testing for the diagnosis of ANCA-associated vasculitis, a systematic review and meta-analysis. Autoimmun Rev.

[6] Emmi G, Bettiol A, Gelain E (2023). Evidence-based guideline for the diagnosis and management of eosinophilic granulomatosis with polyangiitis. Nat Rev Rheumatol.

[7] Grayson PC, Ponte C, Suppiah R (2022). 2022 American College of Rheumatology/European Alliance of Associations for Rheumatology classification criteria for Eosinophilic granulomatosis with polyangiitis. Ann Rheum Dis.

[8] Yates M, Watts RA, Bajema IM (2016). EULAR/ERA-EDTA recommendations for the management of ANCA-associated vasculitis. Ann Rheum Dis.

[9] Mahmood K, Butt NI, Ashfaq F, Aftab S (2023). Eosinophilic granulomatosis with polyangiitis (EGPA): a case report with atypical presentation. Pak J Med Sci.

[10] Kobayashi T, Kanno K, Kikuchi Y (2019). An atypical case of non-asthmatic eosinophilic granulomatosis with polyangiitis finally diagnosed by tissue biopsy. Intern Med.

[11] Kezić A, Ristić S, Životić M, Marković-Lipkovski J, Kovačević S, Naumović R, Ležaić V (2021). Nonasthmatic Churg-Strauss syndrome superimposed on chronic pyelonephritis: a case report. J Int Med Res.

[12] Limgenco-Hipe JR, Manapat-Reyes B (2015). A case of Churg-Strauss syndrome presenting with foot drop. Philippine J Internal Med.

[13] (2025). Eosinophilic granulomatosis with polyangiitis diagnosed in a 55-year old Filipina initially presented as acute polyneuropathy: a case report. https://www.herdin.ph/index.php/component/herdin/?view=research&cid=83658.

[14] Akiyama M, Kaneko Y, Takeuchi T (2021). Rituximab for the treatment of eosinophilic granulomatosis with polyangiitis: a systematic literature review. Autoimmun Rev.

